# Potential resistant mutations within HBV reverse transcriptase sequences in nucleos(t)ide analogues-experienced patients with hepatitis B virus infection

**DOI:** 10.1038/s41598-019-44604-6

**Published:** 2019-05-30

**Authors:** Xiaoman Zhang, Xianli Chen, Meijuan Wei, Chunyu Zhang, Tao Xu, Liguan Liu, Zhengju Xu

**Affiliations:** 1 Clinical Liver Center, the 910th hospital of People’s Liberation Army, Quanzhou, 362000 China; 2 Department of Infectious and Liver Disease, Xiang’an hospital of Xiamen University, Xiamen, 361000 China; 3 Clinical Liver Center, Decheng hospital of Quanzhou Affiliated of Huaqiao University, Quanzhou, 362000 China

**Keywords:** Hepatitis B, Drug regulation

## Abstract

This study was performed to analyze the potential resistant mutations within HBV reverse transcriptase (RT) sequences against nucleos(t)ide analogues (NA). HBV DNA RT region spanning from amino acid 169 to 250 was amplified and sequenced from 435 HBV patients who experienced NA treatment. Among study’s cohort, genotypes B and C infected patients were 55.9% and 44.1%, respectively. Mutations were recorded in 54.7% (238/435) patients at 22 positions. Genotype C displayed significant higher frequency of potential NA resistant mutations than genotype B (63.0% *vs*. 48.1%, *P* = 0.003). Moreover, eight mutation sites, including 180, 181, 191, 200, 202, 221, 229 and 224, in genotype C showed significant higher frequencies than in genotype B. In contrast, mutation at site 236 was more common in genotype B. Notably, 11 mutations at position 169, 202, 250, 173, 180, 200, 207, 214, 237, 242 and 245 coexisted with M204I or V. Substitutions at nine non-classical mutation sites (191, 207, 213, 218, 221, 224, 229, 238 and 242) were detected in patients with virological breakthrough. Particularly, tenofovir (TDF) resistance was observed in one patient undergoing TDF monotherapy and experienced several NA treatment before. These results might provide clinical useful information under antiviral therapy.

## Introduction

Since the discovery of HBV in 1960, chronic HBV (CHB) infection has become major health burden worldwide. Indeed, CHB infection affects 350–500 million people worldwide, which represents approximately 4% of the global population. It is estimated that chronic HBV infection leads to 800,000–1.2 million deaths every year from HBV related consequences, such as cirrhosis, liver failure and liver cancer^1–4^. The prevalence of HBV infection varies in different areas, with the highest number of affected individuals in African countries and Western Pacific regions. In particular, HBV represents one of the top public health priorities in China due to its high extraordinary prevalence, which can reach up to 13% in some regions and ethnic groups. Since the introduction of massive universal HBV vaccination programs in the early 80 s and 90 s, significant improvements have been achieved in HBV infection control, especially in China. However, there are still many challenges in HBV infection control, including higher prevalence in floating population, poor compliance of antiviral therapy, high economic burden and irregular drug use^5^.

To date, ten genotypes (A~J) have been identified worldwide with distinct geographical distributions. In China, genotypes B and C are two predominant ones, which distributed mainly in South and North China, respectively^6^. For suppressing HBV replication and preventing disease progression, five NA have been applied in China: lamivudine (LMV), telbivudine (LdT), adefovirdipivoxil (ADV), entecavir (ETV) and tenofovir (TDF)^7^. These antiviral therapies suppress viral replication without eliminating the virus due to the HBV genome persists in cells as a stable covalently closed circular DNA (cccDNA) for extended lengths of time. As a result, prolonged and often life-long treatments are necessary to control viral replication and reduce the risk of advanced liver diseases such as cirrhosis and liver cancer. Accordingly, there are high chances of emergence of viral resistant mutants^8^. Moreover, a large number of Chinese patients use LAM or LDT over other NA, most due to economic reasons. Regretfully, LAM and LDT have low genetic barrier to develop resistant mutants within HBV reverse transcriptase (RT) region.

RT resistance mutations to NA are generally classified into four categories, including 42 potential NA resistant mutation positions^9–12^. The first category is responsible for reduced treatment susceptibility to antiviral agents. The second category could restore functional defects in the RT activity of HBV caused by primary drug resistance^13–16^. These two categories are known as classical mutations, which include: (i) M204I/V mutation, which associates with LAM or LDT resistance; (ii) N236T or A181T/V mutations, which associate with ADV resistance; (iii) M204V + L180M and either T184A/G/I/L/S or S202G or M250V to develop resistance to ETV^9,14,17^. The third category which named putative antiviral resistance mutation may relate to prolonged NA treatment or replication compensation, such as S53N, T54N and L82M. The fourth category could be found before NA therapy, such as T38A, Y124H and D134E^12^. The latter two categories are known as non-classical mutations. In contrast to classical mutations, most of the mutations in the third and fourth categories lack phenotypic resistance data, and the clinical significance is not clear.

Several reports have revealed the clinic differences between genotypes A and D, similarly to genotypes B and C^18-21^. What’s more, many studies have demonstrated the correlations between HBV genotypes and antiviral efficacy (including IFN-α, NA therapies) and prognosis of disease^20,22,23^. However, the association between genotypes of HBV and NA resistance mutations has been limited studied now, which might due to the complex of NA usage in real-life clinic practice.

In this study, the treatment information of 435 HBV infected patients was recorded in detail. We observed characters of potential NA resistant mutations in HBV RT region among these patients, including differences between genotypes B and C, some interesting non-classical mutations and the evolution of HBV mutants.

## Materials and Methods

### Patients

A total of 435 HBV infected patients, who experienced treatment of NA, were enrolled in the present study. These patients attended the 910th Hospital (Quanzhou, China) during the period January 2012 to August 2017. Genotypes B and C infected patients were 55.9% (243/435) and 44.1% (192/435), respectively (Table 1). HBV was isolated from serum samples at the time point when HBV drug resistance was suspected by clinical doctor, which included any of the following scenarios: (1) Serum HBV DNA level did not decrease after three months of antiviral therapy; (2) Serum HBV DNA level was still more than 500 copies/ml after one year of continuous antiviral therapy; (3) Viral breakthrough appeared. A formal consent was collected from each patient, and the study was ratified by the Ethics Committee of the 910th Hospital. The use of serum samples involved in following experiments was in accordance with the Ethics Committee of the 910th Hospital too.

**Table 1:** Patient characteristics.

Characteristic	Genotype B (n = 243)	Genotype C (n = 192)	*P-*value
Male/female gender (% males)	205/38 (84.4)	155/37 (80.7)	0.350
Median age, (range)	34 (12–73)	41 (14–78)	<0.001
Mean HBV DNA, log_10_ IU/ml (SD)	5.4 (1.4)	5.5 (1.4)	0.982
Positive/negative HBeAg status (% positive)	189/54 (77.8)	150/42 (78.1)	0.976
Mean ALT level, U/L(SD)	160.0 (246.4)	166.8 (275.8)	0.929
Mean AST level, U/L(SD)	98.0 (192.0)	124.6 (295.9)	0.113

These patients were divided into sixteen groups based on their treatment with median therapy duration of 36.0 months (range 1.0 to 156.0 months) (Table 2). Patients with serum HBV DNA of <500 copies/ml and had liver damage caused by hepatitis A, C, D and E, or other factors were excluded. The clinical diagnosis of chronic hepatitis B and the definition of virological breakthrough were according to EASL 2012 clinical practice guidelines^1^.

**Table 2:** Comparison of NA usage and potential NA resistant mutations between genotypes B and C groups.

Treatment	No. cases (%)		*P-*value	Therapy duration, months (mean, SD)		*P-*value	Potential NA resistant mutations (n, %)		*P-*value
	Genotype B (n = 243)	Genotype C (n = 192)		Genotype B (n = 243)	Genotype C (n = 192)		Genotype B (n = 243)	Genotype C (n = 192)	
L-Nucleosides as initial therapy									
LAM	37 (15.2)	27 (14.1)	0.838	35.0 (30.3)	31.0 (19.5)	0.837	19 (51.4)	23 (85.2)	0.326
LDT	19 (7.8)	10 (5.2)	0.373	17.0 (8.9)	23.0 (20.5)	0.429	11 (57.9)	7 (70.0)	0.813
L-Nucleosides→ADV	30 (12.5)	17 (8.7)	0.313	54.0 (32.2)	61.0 (45.0)	0.791	11 (36.7)	12 (70.6)	0.053
L-Nucleosides→ETV	7 (2.8)	12 (6.1)	0.141	48.0 (33.5)	48.0 (32.7)	0.871	7 (100.0)	7 (58.3)	0.147
L-Nucleosides→ADV/ETV	15 (6.3)	15 (7.8)	0.632	74.0 (37.6)	78.0 (45.3)	0.853	10 (66.7)	12 (80.0)	0.680
ADV as initial therapy									
ADV	23 (9.5)	29 (15.1)	0.090	25.0 (19.0)	51.0 (22.1)	0.563	9 (39.1)	15 (46.9)	0.768
ADV→ L-Nucleosides	8 (3.5)	5 (2.6)	0.893	50.0 (28.9)	39.0 (19.0)	0.429	2 (25.0)	2 (40.0)	0.962
ADV→ETV	12 (4.9)	4 (2.08)	0.188	32.0 (10.2)	38.0 (17.2)	0.758	2 (16.7)	0 (0.0)	0.640
ADV→ L-Nucleosides/ETV	2 (0.7)	7 (3.5)	0.086	56.0 (33.9)	62.0 (10.7)	—	2 (100.0)	7 (100.0)	0.182
ETV as initial therapy									
ETV	56 (23.0)	32 (16.7)	0.127	43 0.0(15.3)	26.0 (20.2)	0.229	21 (37.5)	13 (40.6)	0.951
ETV→ADV	14 (5.6)	4 (2.08)	0.095	56.0 (43.1)	63.0 (24.8)	0.906	10 (71.4)	2 (66.7)	0.593
ETV→ L-Nucleosides	10 (4.2)	5 (2.6)	0.553	63.0 (24.8)	46.0 (17.1)	0.819	8 (80.0)	2 (40.0)	0.333
ETV→ L-Nucleosides/ADV	0 (0.0)	3 (1.7)	0.170	0	52.0 (21.0)	—	—	3 (100.0)	—
Other NAs as initial treatment									
LAM + ADV	8 (3.5)	13 (7.0)	0.146	47.0 (36.3)	24.0 (12.6)	0.242	3 (37.5)	7 (53.8)	0.648
LAM + ADV→ETV	0 (0.0)	2 (0.9)	0.378	0	42.0 (19.5)	—	—	2 (100.0)	—
ETV + ADV	2 (0.7)	7 (3.5)	0.086	45.0 (15.4)	39.0 (25.1)	—	2 (100.0)	7 (100.0)	0.182

L-Nucleosides: LAM or LDT.

### Laboratory tests

Alanine aminotransferase (ALT) and Aspartate aminotransferase (AST) levels were determined using the AU680 Chemistry System (Beckman Coulter, Brea, CA, USA), with a normal value ≤40 IU/L. HBeAg was detected using a Roche cobas e601 (Roche Diagnostics, Indianapolis, IN). HBV DNA quantification was performed using RT-PCR, according to manufacturer’s instructions (Shanghai Fosun Industrial Limited by Share, Ltd., Shanghai, China), followed by real-time PCR using the Mx3000P system (Agilent Technologies, Santa Clara, CA, USA), with an HBV detection limit of 500 copies/ml.

### HBV RT region sequencing

The HBV DNA RT region spanning from rt169 to rt250 was amplified by real-time PCR using the Mx3000P system (Agilent Technologies, Santa Clara, CA, USA). PCR product-based direct sequencing were actualized by ABI PRISM 310 Genetic Analyzer (Applied Biosystems, Waltham, MA, USA). Nucleotide sequences were analyzed using the DNA Star 5.0 and MEGA 4.0 software. Mutations sites in HBV RT region were determined according to the definition of potential NA resistant mutations^12,14,24,25^^.^

### Statistical analysis

Data were statistically analyzed using Prism 5.0 (GraphPad Software, La Jolla, CA, USA). Results were expressed as mean ± standard deviation (SD) or median. Quantitative values were analyzed using Student’s *t*-test. Qualitative values were analyzed using chi-squared test. *P* < 0.05 was considered statistically significant.

### Informed consent statement

Written informed consents have been obtained from each patient, including approval of the treatment, willingness to publish details of the case, and consent to use of blood samples for the tests involved in this study.

## Results

### Patient characteristics

The demographic, biochemical, virological and therapeutic characteristics of patients are displayed in Table 1. Among the 435 patients enrolled in this study, 55.9% (243/435) patients had HBV genotype B infection, while 44.1% (192/435) patients had HBV genotype C infection. 82.8% (360/435) patients were male and 17.2% (38/435) patients were female, and the overall median age was 38 years old. Furthermore, a total of 77.9% (339/435) patients were tested positive for HBeAg. There was no significant difference in gender, serum HBV DNA level, HBeAg positive rate, serum level of ALT and AST between genotypes B and C groups. But, a significantly higher age were found in genotype C group (*P* < 0.001).

### Comparison of NA therapy and potential NA resistant mutations

In real-life clinic practice, the antiviral therapy strategies were very complex. In order to minimize the bias of NA usage between genotypes B and C infected patients, sixteen treatment regimens were summarized in Table 2: LAM, LDT, ADV and ETV single, sequential or combination treatments. For example, treatment of L-Nucleoside → ADV represented LAM or LDT as initial therapy followed by switch to or add on ADV. Follow-up treatment might also include switching to or add on L-Nucleoside but not ETV. Notably, TDF was used as antiviral drug in only six patients. To simplify the treatment groups, these six cases were classified in sixteen categories mentioned above, according to the usage of other NA drugs.

Table 2 showed that there was no significant difference in distribution rates of genotypes, therapy duration time and prevalence of potential NA resistant mutations between genotypes B and C groups under various antiviral regimens.

### Comparison of potential NA resistant mutation sites

Within the RT region that spans from amino acid I169 to M250, 26 positions associated with potential resistance to NA treatment were included. Among 435 patients, 238 samples (54.7%, 238/435) with 22 sites were found to be mutated, including 9 classical mutation sites (categories 1 and 2) and 13 non-classical mutation sites (categories 3 and 4) (Table 3). Notably, mutations at position 194, 215, 217 and 233 were not found. In general, the potential NA resistant mutation rates was significantly higher in genotype C group than in genotype B group (121/192, 63.0% *vs*. 117/243, 48.1%, *P* = 0.003).

**Table 3:** Prevalence of potential NA resistance mutations between I169 to M250 in HBV RT region.

Mutation category	Mutation Types	Genotype B (n = 243) (%)	Genotype C (n = 192) (%)	*P-*value
1. Primary drug resistance mutation	I169M	1 (0.4)	0 (0.0)	0.906
	A181T/V/S/G	10 (4.1)	22 (11.5)	0.006
	T184K/L/I/T/P/S/A	6 (2.5)	9 (4.7)	0.320
	S202G	2 (0.8)	10 (5.2)	0.013
	M204I/V	84 (34.6)	82 (42.7)	0.102
	N236T/V/K/I/A	17 (7.0)	4 (2.1)	0.032
	M250L/V	7 (2.9)	1 (0.5)	0.144
2. Compensatory mutation	V173L/M	1(0.4)	3 (6.8)	0.458
	L180M/Q	31 (12.8)	50 (26.0)	<0.001
3. Putative NAr mutation	V191I	0 (0.0)	6 (3.1)	0.018
	A200V	0 (0.0)	9 (4.7)	0.002
	V207I/L/M/F	4 (1.6)	5 (2.6)	0.720
	S213T	8 (3.3)	2 (1.0)	0.218
	V214A	0 (0.0)	2 (1.0)	0.378
	E218D	1 (0.4)	0 (0.0)	0.906
	Y/F221F/Y	2 (0.8)	15 (7.8)	<0.001
	L229F/M/V/W	6 (2.5)	18 (9.4)	0.003
	P237S/H	1 (0.4)	2 (1.0)	0.835
	H/N238N/H//L/Q/R/A/S/T	5 (2.1)	10 (5.2)	0.128
	Y245F	0 (0.0)	1 (0.5)	0.906
4. Pretreatment mutation	V/I224I/V	0 (0.0)	16 (8.3)	<0.001
	R242D	1 (0.4)	0 (0.0)	0.906

In detail, genotype C infected patients had higher mutation frequencies, compared with genotype B infected patients at eight sites, including 180, 181, 191, 200, 202, 221, 229 and 224. In contrast, mutation rates at site 236 were much higher in genotype B infected patients than in genotype C infected patients (Table 3). In addition, mutations at 5 sites (191, 200, 214, 245 and 224) were found only in genotype C infected patients.

### Comparison of M204, A181 and N236 mutation patterns

**Figure 1:**
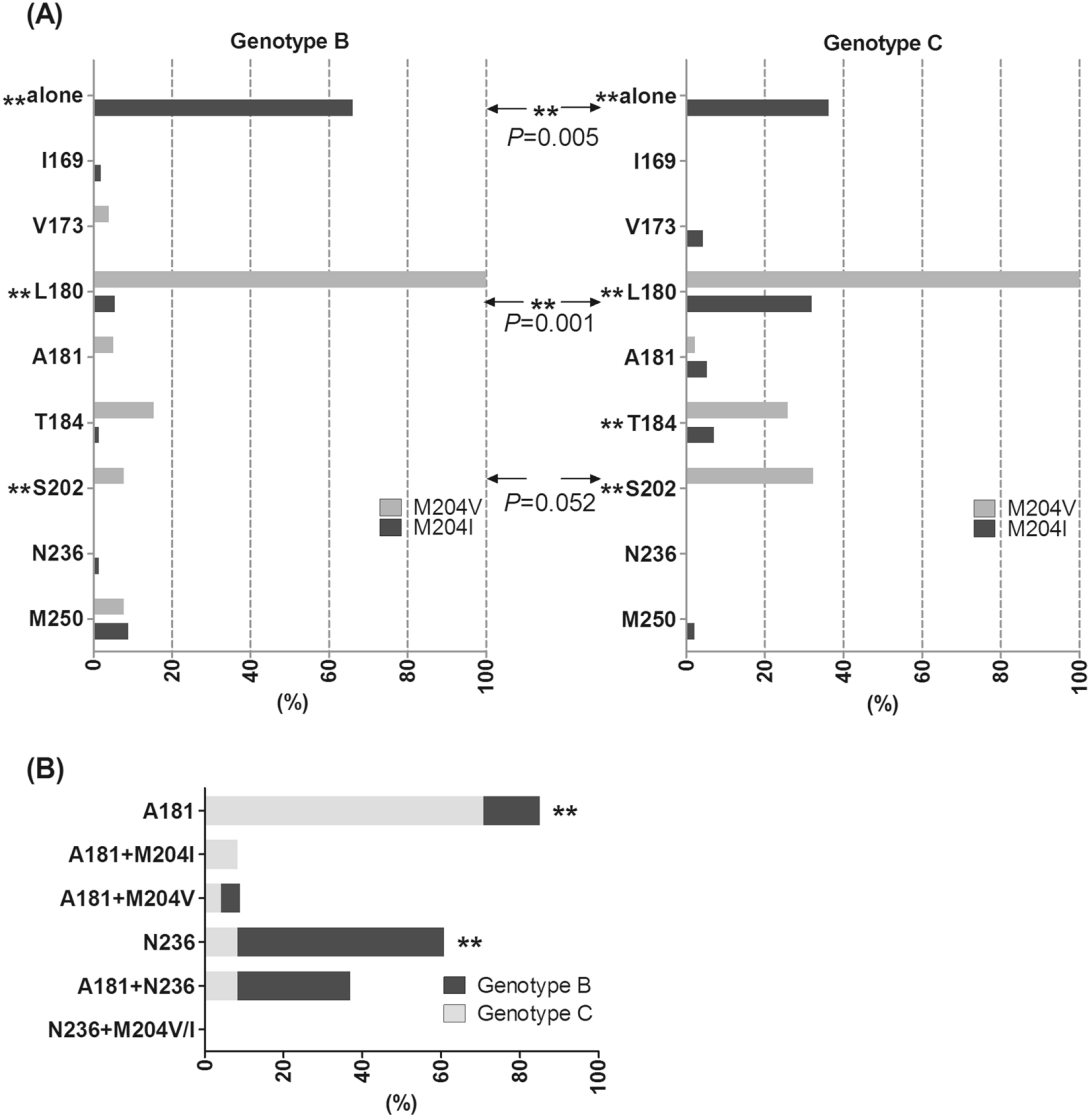
Comparison of M204, A181 and N236 mutation patterns in genotypes B and C. (**A**) The prevalence of mutations associated to M204I or V. The asterisk on the left of the mutation sites indicate significant differences in the mutations associated to M204I or V. The arrows indicate significant differences in M204 mutation patterns between genotype B and C. (**B**) Main mutation patterns of A181 and N236.*P < 0.05; **P < 0.01.

Mutations at position 204 was the most frequent recorded in this study (38.16%, 166/435), followed by mutations at position 180 (18.62%, 81/435), 181 (7.36%, 32/435), 229 (5.52%, 24/435) and 236 (4.83%, 21/435). Only V or I was detected as mutant amino acids at site M204, in only six cases mixed with V and I. Samples included mutations of M204I in genotypes B and C were 56 and 47, respectively. Similarly, samples including mutations of M204V in genotype B and C were 26 and 31, respectively. Notably, 11 mutations at position 169, 202, 250, 173, 180, 200, 207, 214, 237, 242 and 245 coexisted with M204I or V (data not shown). Figure 1A showed the association of classical mutation sites to M204I or V in genotypes B and C, respectively. No single mutation of M204V was detected in both genotypes. However, in genotype B, single mutation of M204I was much more frequent in genotype B than in genotype C (37/56, 66.1% *vs*. 17/47, 36.2%, *P* = 0.005). In contrast, the prevalence of mutation pattern of L180 associated to M204I in genotype C was significantly higher than in genotype B (15/47, 31.9% *vs*. 3/56, 5.4%, *P* = 0.001).

As we know, there was a completely opposite mutation rates at sites 181 in genotype C and 236 in genotype B, mentioned above in Table 3. Figure 1B further analyzed the main mutation patterns of these two sites without considering non-classical mutations. Patients included A181 or (and) N236 mutations in genotype B were 21. In genotype C infected patients, 24 cases were observed having A181 or (and) N236 mutations. It showed that, single mutations of A181 rather than combined mutation patterns of A181 in genotype C was significantly higher than in genotypes B (17/24, 70.8% vs. 3/21, 14.3%, *P* < 0.001). However, single mutations of N236 in genotype B was more frequently (11/21, 52.4% vs. 2/24, 8.3%, *P* = 0.003).

### Non-classical mutational patterns of patients with virological breakthrough and without classical mutations

As we known, there is little understanding of non-classical mutations (categories 3 and 4). In this study, non-classical mutations were observed in 29 patients who suffering from virological breakthrough and without classical mutations (Table 4) at 9 sites (V191I, V207I/M, S213T, E218D, F221Y, I224V, L229V, N/H238 and R242D). These sites could be classified into the following groups: V191I and V207I/M were detected in cases experienced ADV treated; F221Y were found in cases experienced LAM treated; S213T, I224V and H/N238 seemed to be the sharing mutation sites of LAM, ADV and ETV. Because theses mutations could be caused by the single treatment of LAM, ADV and ETV respectively; E218D was identified in LAM switched to ADV therapy; R242D was found in ADV switched to ETV therapy.

**Table 4:** Non-classical mutation patterns of patients with virological breakthrough and without classical mutations.

Mutation type	Genotype	Treatment	Mutation type	Genotype	Treatment
V191I	C	ADV	F221Y + L229V	C	LAM → ETV → ADV
V191I	C	ADV	F221Y + L229M	C	LAM + ADV
V191I	C	ETV + ADV	F221Y + I224V + N238H	C	LAM + ADV → ADV
V207I/M + L229M	C	ADV	I224V	C	ADV
S213T	B	ADV	I224V	C	LAM
S213T	B	ETV	I224V	C	ETV
S213T	B	ETV	I224V	C	LAM
S213T	B	ADV	I224V + N238A	C	ADV
S213T	B	LAM	I224V + N238T	C	LAM
S213T	C	ADV	H238Q	B	LAM → LAM + ADV
S213T	B	LAM + ADV → ADV	H238N	B	ETV → LAM
S213T	B	ETV	N238T	C	ETV
E218D	B	LAM → ADV	H238Q	B	ADV
F221Y	C	LAM	R242D	B	ADV → ETV
F221Y	C	LAM + ADV			

Interestingly, all mutations of L229 were associated to other sites in this study. Out of 24 mutations of L229 (Table 3), 18 were associated to M204I or V; 2 coexisted with F221Y (Table 4); 2 were associated to A181T; 2 were associated to V207M (Table 4) and N236V, respectively. These non-classical mutations might be closely related to clinical resistance observed in this study.

### Evolution of HBV mutants throughout the years of antiviral therapy

**Figure 2:**
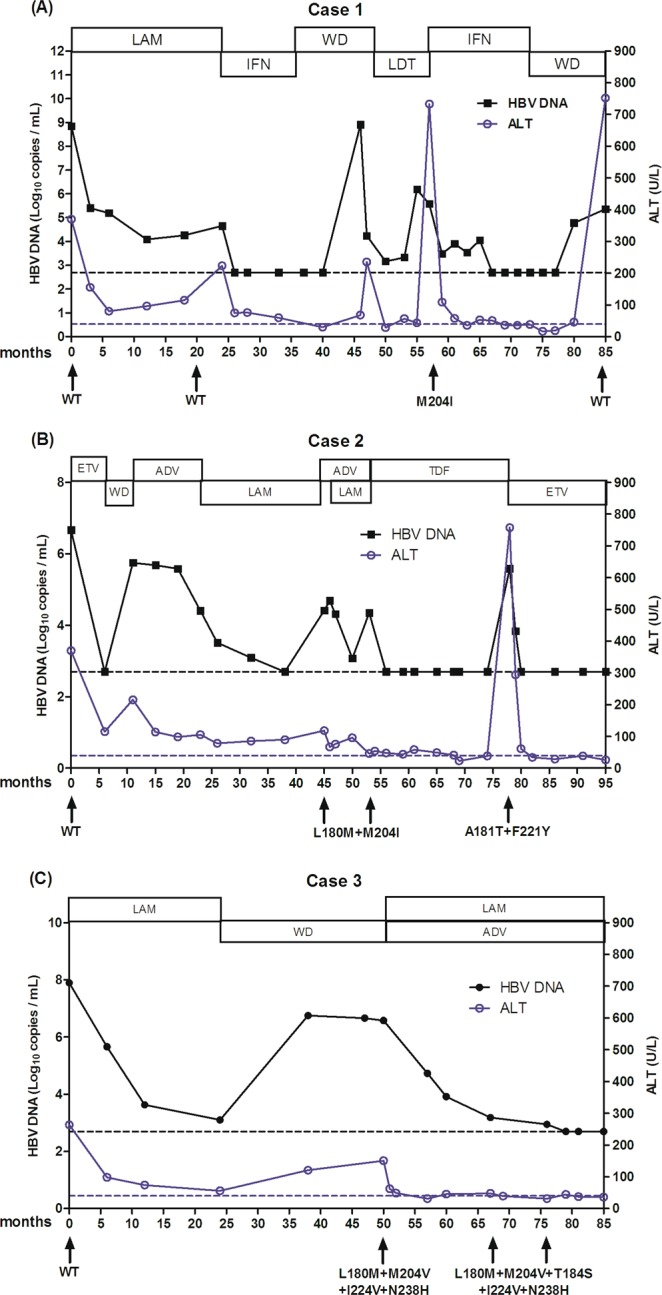
(**A**–**C**) Evolution of HBV mutants with serum ALT and HBV DNA level in three patients during the long-term of antiviral therapy. Black dotted lines represent detection limit of serum HBV DNA level (500 copies/mL). Blue dotted lines represent upper normal limit of serum ALT level. WD: withdraw; WT: wild-type.

Finally, multiple mutation patterns were observed in three patients throughout the years of antiviral therapy in current cohort (Fig. 2). In case one, mutation patterns changed from wild-type to M204I and back to wild-type again during 85 months of antiviral therapy (Fig. 2A). Interestingly, mutation pattern of A181T associated to F221Y was detected after 22 months of TDF monotherapy with virological and biochemical breakthrough in case two (Fig. 2B). In case three, multiple mutation patterns were observed from L180M + M204V + I224V + N238H to L180M + M204V + T184S + I224V + N238H under LAM and ADV combined therapy, which developed resistance to ETV. Fortunately, the serum level of HBV DNA and ALT felled to normal under the continuous treatment of LAM and ADV (Fig. 2C). However, the resistance surveillance and clinical features needed further observation. Obviously, the emergence of recovery mutation and multiple mutation patterns was closely related to drug therapy schedule and clinical features.

## Discussion

The determination and characterization of potential NA resistant mutations in the HBV RT region including the differences between genotypes are quite necessary to tailor therapy strategy during long-term antiviral treatment for HBV patients, especially in China.

Genotypes B and C are two dominant ones in South and North China, respectively. Limited data on association of genotypes B and C with potential NA resistant mutations were found, partly due to small samples size and complex drug usage in real-life clinical therapy. The present study, a relatively large samples size were enrolled in and deviation of NA usage were balanced minimized. The genotype B infected patients (55.9%) were much higher than genotype C infected patients (44.4%), which was congruent with other studies^26,27^. In present study, the proportion of potential NA resistant mutations were 54.7% (238/435), which was in middle level among other regions of China^28–32^. Genotype C displayed higher mutation rates than genotype B (63.0% *vs*. 48.1%). In addition, genotypes showed different inclinations to develop certain NA drug resistance associated mutations in this study. For L-nucleosides resistance, M204I/V was the most critical mutation site. Unlike M204V, variant of M204I was highly resistant to LDT^7^. Zollner *et al*.^17^ reported that M204I appeared in a minority of resistant strains in genotype A and usually existed as an isolated one. By contrast, it is selected more frequently than M204V in genotype D. Our data showed no significant difference was found in frequencies of M204I or V mutation. When taking into account the associated sites, M204I mutation itself was more prevalent in genotype B, while L180 + M204I was more common in genotype C (Fig. 1A), which consistent with Zhang *et al*.^29^ and Guo *et al*.^33^. In both genotypes L180 was practically necessary for M204V. Overall, there was maybe little difference in L-nucleosides resistance between genotype B and C. However, L180 + M204I formed the basis of ETV resistance, which together with the higher mutation rates at other three ETV-resistant mutation sites (180, 200 and 202) in genotype C (Table 3), contributed to more favorable for further evolution towards ETV resistance in genotype C. For ADV resistance, genotypes B expressed higher frequencies at mutation site N236. Yet A181 mutations was more common in genotype C (Table 3, Fig. 1B), which could develop multidrug resistance. The underlying mechanism of this peculiar mutations and mutation patterns between genotypes needed further studied in future. It is profitable to cognizing the higher tendency of ETV resistance and multidrug resistance in genotype C in clinical antiviral practice.

Non-classical mutations (categories 3 and 4) in HBV RT region were unvalued in clinical practice, which may due to relatively low frequencies, lack of phenotypic resistance data and obscure clinical significance. Among 435 patients, non-classical mutations were detected at nine sites (V191I, V207I/M, S213T, E218D, F221Y, I224V, L229V, N/H238 and R242D) with virological breakthrough and without classical mutations (Table 4). Yang *et al*.^34^ reported that V191I was observed after 6 months of treatment with ADV, similar with that in present study (Table 4). Interestingly, *in vitro* test, the V191I mutant remained sensitive to ADV. Zöllner *et al*.^35^ found that V207I restored viral replication fitness in LAM-resistant HBV, exhibiting YMDD mutations, and indicating the compensatory function of the rtV207I mutation. Other studies have suggested that V207I moderately decreased the sensitivity to LAM and ADV^36,37^. Mirandola *et al*.^38^ reported that F221Y was found under ADV therapy in genotype A, Lee *et al*.^39^ reported F221Y was detected in one case suffering viral breakthrough under TDF monotherapy following an initial unsuccessful LAM and ADV combination therapy. In this study, F221Y was observed in cases not only under ADV therapy but also under LAM treatment (Table 4). In another case in this study, the patient experienced viral breakthrough after 22 months of TDF monotherapy with the mutant HBV bearing mutation pattern of A181T + F221Y (Fig. 2B). These data indicated that F221Y might associate with LAM, ADV and TDF treatment. In current study, mutation of L229 was not a single mutation site: 75% mutations of this site associated to M204I or V. Ji *et al*.^40^ and Kwon *et al*.^41^ showed that L229F and L229V might act as a compensatory mutation for M204I, although the susceptibility to LAM was not reduced, similarly to another study. It revealed that L229W did not change the susceptibility to NA in one vitro mutational analysis^42^. In present study, besides bases of phenylalanine (F) and valine (V), isoleucine (I) and methionine (M) were also recorded at site 229. These two residues had not been reported yet and needed to further research. The mutation at amino acid 238 has been reported may be associated with ADV resistance *in vitro*^43,44^. Our data showed that mutation at 238 seemed to be caused by single LAM, ADV and ETV respectively. Kwon *et al*.^45^ reported that single mutation of N238H did not affect replication under clevudine treatment, while the replication efficiency was significantly reduced by N238H + K333N double mutant *in vitro*. Yamada *et al*.^46^ proved that N238H mutation had little effect on ETV resistance. Zhong *et al*.^47^ considered that N238H variant did neither influence the susceptibilities to LAM or ADV nor weak the viral replication efficiency *in vitro*. It may be the polymorphism of HBV rather than resistance mutations. These non-classical mutations sometimes occurred alone, sometimes combined with other mutations. The influence on drug resistance was really complex and puzzled, which needed extensive research in future.

Since the NA treatment for CHB needed be used for a long period of time, which could lead to the emergence of HBV multiple mutation patterns. An appropriate therapy should be used with another agent which does not share cross resistance mutations to adapt the certain mutants^7^. TDF showed high antiviral efficacy and high genetic barrier to resistance not only in LAM resistance, ADV resistance, ETV resistance, but also in poor viral response patients. So far, rarely resistant mutation to TDF has been identified. Sheldon *et al.*^48^ reported that A194T conferred a reduced susceptibility to TDF *in vitro* in two HBV and human immunodeficiency virus (HIV) co-infected patients. In addition, mutations of A181T/V and N236T showed intermediate susceptibility to TDF^7^. Lee *et al.*^39^ reported that an combined mutation pattern of L80M, L180M, M204V/I, A200V, F221Y, S223A, T184A/L, R153Q, and rtV191I was detected at the time of virological and biochemical breakthrough emerged during TDF monotherapy, following an unsuccessful LAM, ETV and ADV sequential or combined treatment. Notably, in present study, the mutation pattern of A181T associated with F221Y was observed in one case after 22 months of TDF monotherapy with virological and biochemical breakthrough. Regretfully, mutant strains could not be detected in detail, due to the limitations of direct sequencing used in this study. Even so, the main mutants of A181T and F221Y might closely relate to TDF therapy. Hence, monitoring of TDF resistance in patients experienced several NA drugs during the whole process of antiviral therapy is quite necessary.

## Data Availability

The data used to support the findings of this study are available from the corresponding author on reasonable request.
